# Alterations in cognitive function and blood biomarkers following transcranial direct current stimulation in patients with amyloid positron emission tomography-positive Alzheimer’s disease: a preliminary study

**DOI:** 10.3389/fnins.2023.1327886

**Published:** 2023-12-21

**Authors:** Jinuk Kim, YoungSoon Yang

**Affiliations:** ^1^Center for Neuroscience Imaging Research, Institute for Basic Science (IBS), Suwon, Republic of Korea; ^2^Department of Neurology, Soonchunhyang University College of Medicine, Cheonan Hospital, Cheonan, Republic of Korea

**Keywords:** transcranial direct current stimulation (tDCS), Alzheimer’s disease (AD), cognitive function, Aβ oligomers, blood biomarker

## Abstract

**Introduction:**

Alzheimer’s disease (AD), the most common form of dementia, is characterized by progressive cognitive decline. To address this, we conducted a randomized, double-blinded, sham-controlled study to investigate the therapeutic potential of transcranial direct current stimulation (tDCS) on patients with amyloid positron emission tomography (PET)- positive AD.

**Methods:**

Participants already undergoing pharmacological treatment and testing positive for amyloid PET were divided into Active-tDCS (*n* = 8) and Sham-tDCS (*n* = 8) groups. For 12 weeks, participants or their caregivers administered daily bi-frontal tDCS (YMS-201B+, Ybrain Inc., Seongnam, Korea) at home (2 mA, 30 min). Pre- and post-intervention assessments included neuropsychological tests and blood sample measurements for oligomerized beta-amyloid.

**Results:**

The Active-tDCS group demonstrated significant improvements in cognitive domains such as language abilities, verbal memory, and attention span and in frontal lobe functions compared to the Sham-tDCS group. Furthermore, the Active-tDCS group showed a marked reduction in post-intervention plasma Aβ oligomerization tendency level, suggesting changes in pivotal AD-associated biomarkers.

**Discussion:**

Our results emphasize the potential therapeutic benefits of tDCS for mild AD patients with amyloid PET positivity and stress the urgency for broader research, considering the global challenges of dementia and the need to pursue innovative therapeutic strategies.

## 1 Introduction

Republic of Korea is transitioning into a ‘super-aged society’ faster than any other country, and requires a systematic approach to manage age-related diseases. Dementia is a serious condition that primarily affects the elderly population. The substantial costs associated with the treatment and management of this disease place a heavy burden on both healthcare expenditures and the national healthcare budget. When early detection and treatment are delayed, the burden on the national healthcare budget progressively increases, demonstrating that dementia significantly impacts these resources (Wimo et al., 2013).

Current therapeutic strategies for dementia, and particularly Alzheimer’s disease (AD), are centered around addressing the degeneration of cholinergic nerves that progresses from the basal nucleus of Meynert toward the hippocampus or temporal lobe (Cummings and Benson, 1987). This degeneration has been reported to cause a decrease in acetylcholine level in the brain, a factor associated with cognitive impairment, behavioral disorders, and functional decline. Based on these findings, acetylcholinesterase inhibitors (AChEIs) have been developed and used as a primary treatment for Alzheimer’s disease (Zuin et al., 2022). These drugs work by preventing the breakdown of acetylcholine, a vital neurotransmitter involved in memory and learning, increasing it concentration in the brain. This treatment helps to alleviate the symptoms of Alzheimer’s, providing patients with improvements in cognitive function and quality of life (Tanaka et al., 2022). However, while these treatments can provide symptomatic relief, they do not halt the disease progression. The limitations of current Alzheimer’s treatments underscore the critical need for innovative therapeutic approaches that not only manage symptoms, but also address the underlying disease mechanisms to potentially slow or halt disease progression.

While AChEIs are the frontline treatment for Alzheimer’s disease, they are not without side effects. Patients may experience gastrointestinal disturbances, bradycardia, fainting, and sleep disruptions (Mohammad et al., 2017). Given the concerns with AChEIs, there is a need for complementary treatments. Transcranial Direct Current Stimulation (tDCS) is emerging as a promising solution.

Treatment with tDCS aims to improve cognitive functions by specifically targeting and modulating the activity of damaged or underactive brain neurons. By applying direct electrical currents, tDCS capitalizes on the brain’s inherent ability to change and adapt over time, a phenomenon known as neuroplasticity. This capacity of the brain allows it to reorganize and form new neural connections, potentially aiding in the restoration and enhancement of cognitive functions (Flöel, 2014). Functional brain imaging studies, particularly those using Positron Emission Tomography (PET) to detect brain activity, have observed decreased glucose metabolism in the temporal lobe, parietal cortex, and hippocampus of AD patients (Arnáiz et al., 2001). Notable decreases in glucose metabolism have been reported in the posterior cingulate and the ventro-lateral cortex of the prefrontal lobe (Drzezga et al., 2003). Additionally, hyperactivity in bilateral prefrontal areas are evident compared to healthy controls (Meinzer et al., 2015). Many studies have aimed to improve cognition in patients with Alzheimer’s disease and dementia by attaching tDCS electrodes to the bilateral prefrontal regions to stimulate prefrontal function (Ciullo et al., 2021).

Amyloidosis in the brain is a hallmark feature of AD and has been introduced as a recent diagnostic criterion (McKhann et al., 2011). From disease discovery, researchers believed that amyloid plaques (consisting mainly of β 1–40 and β 1–42) were central to AD pathophysiology. However, more recent research suggests that it is not the larger fibril forms of these plaques, but the smaller, soluble aggregates termed ‘Aβ oligomers’ that play a more direct role in disease progression (Gong et al., 2003; Hayden and Teplow, 2013). Most of the blood-based AD biomarkers currently in use are safe, simple, and cost-effective; however, they are limited in clinical application as they are not directly associated with the pathological mechanisms of AD. However, recent advancements allow reliable detection and quantification of the Aβ oligomer that has passed through the blood–brain barrier and is present in the plasma (Babapour Mofrad et al., 2021; Pyun et al., 2021). This suggests that, unlike invasive or expensive methods such as the concentration of Amyloid Beta (Aβ42) in the cerebrospinal fluid (CSF) – which is used as an amyloid biomarker – or amyloid PET imaging, there is potential for a simple alternative in measuring Aβ oligomer in the plasma for diagnosing AD, understanding its pathological mechanism, and conducting drug research.

In this preliminary study, our objective is to investigate the potential cognitive advantages of tDCS for patients with amyloid PET-positive AD who are already receiving pharmacological treatment. After 12 weeks of tDCS application, we assess improvements in cognitive abilities and investigate changes in Aβ oligomer level in the plasma, seeking to understand the mechanism of tDCS in AD.

## 2 Methods

### 2.1 Participants

**Figure 1:**
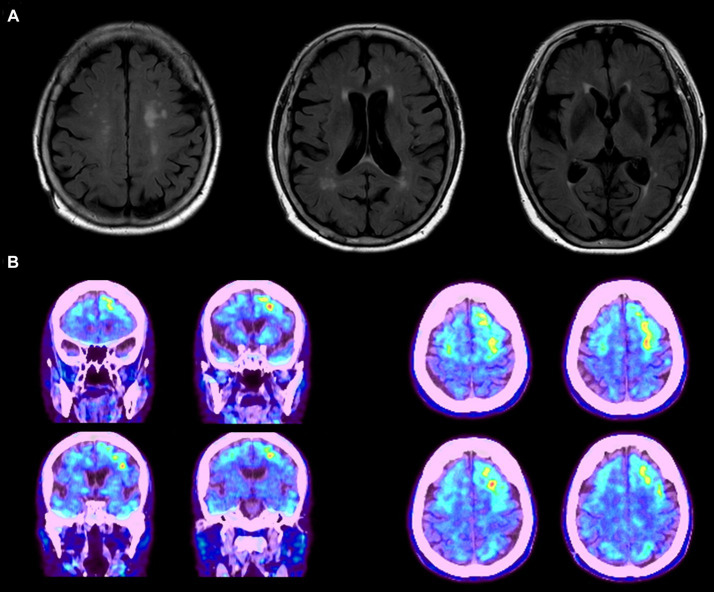
**(A)** MRI data of the proband patient displaying diffuse cortical atrophy and enlarged ventricles. **(B)** Abnormal amyloid deposits observed in the frontal, temporal, and occipital lobes, with predominant accumulation on the left side. The images represent data from a single patient as an example.

Participants included in this study visited Cheonan Hospital of Soonchunhyang University Medical School from January 2021 to July 2022, with memory decline complaints. They were diagnosed with Alzheimer’s dementia based on the criteria of the National Institute of Neurologic and Communicative Disorders and Stroke-Alzheimer Disease and Related Disorders Association (NINCDS-ADRDA) (McKhann et al., 1984). Among these, only those with positive amyloid PET findings and classified as having mild Alzheimer’s dementia symptoms (Clinical Dementia Rating Scale: CDR = 0.5 or CDR = 1) were selected (Figure 1). All participants had to have been on a stable medication regimen without any changes in dosage for at least 3 months. Patients were excluded from the study if they had any of the following conditions: (1) A diagnosis of brain tumors or encephalitis, (2) a psychiatric disorder diagnosed based on the Diagnostic and Statistical Manual of Mental Disorders (DSM-IV) criteria within the 2 years leading up to study commencement, (3) a manifestation of severe depression, as evidenced by a Hamilton Depression Rating Scale (HAM-D) score exceeding 18, (4) other neurological disorders including but not limited to Parkinson’s disease, Huntington’s disease, or normal-pressure hydrocephalus, (5) medical conditions known to induce cognitive impairments, such as liver disease, kidney disease, or thyroid disorders, (6) a known history of alcohol or drug addiction within the previous 2 years, and (7) any physical disabilities that would prohibit successful completion of the tDCS procedure. Demographic characteristics of all participants were recorded. To evaluate cognitive state and severity of AD, we utilized the Korean version of the Mini-Mental State Examination (K-MMSE) by Kang (2006) and the CDR (Morris, 1997).

### 2.2 Experimental protocol

This study was designed as a single-center, double-blind, randomized, sham-controlled pilot study. All participants underwent physical and neurological examination by a neurology specialist. Before and after the intervention, participants underwent a neuropsychological examination conducted by the same neuropsychological examiner. All participants underwent an MRI scan, and blood was collected for blood biomarker analysis. A total of 16 patients diagnosed with mild AD (CDR = 0.5 or 1) was enrolled in this clinical trial. Through random assignment, participants were divided into experimental (Active-tDCS, *n* = 8) and control (Sham-tDCS, *n* = 8) groups. Each group underwent a total of 84 sessions of tDCS intervention, lasting for 30 min daily over a span of 12 weeks. This study was conducted in accordance with the Declaration of Helsinki, and the protocol was approved by the Institutional Review Board of Soonchunhyang University Cheonan Hospital (IRB No. 2021-05-034).

### 2.3 tDCS administration

**Figure 2:**
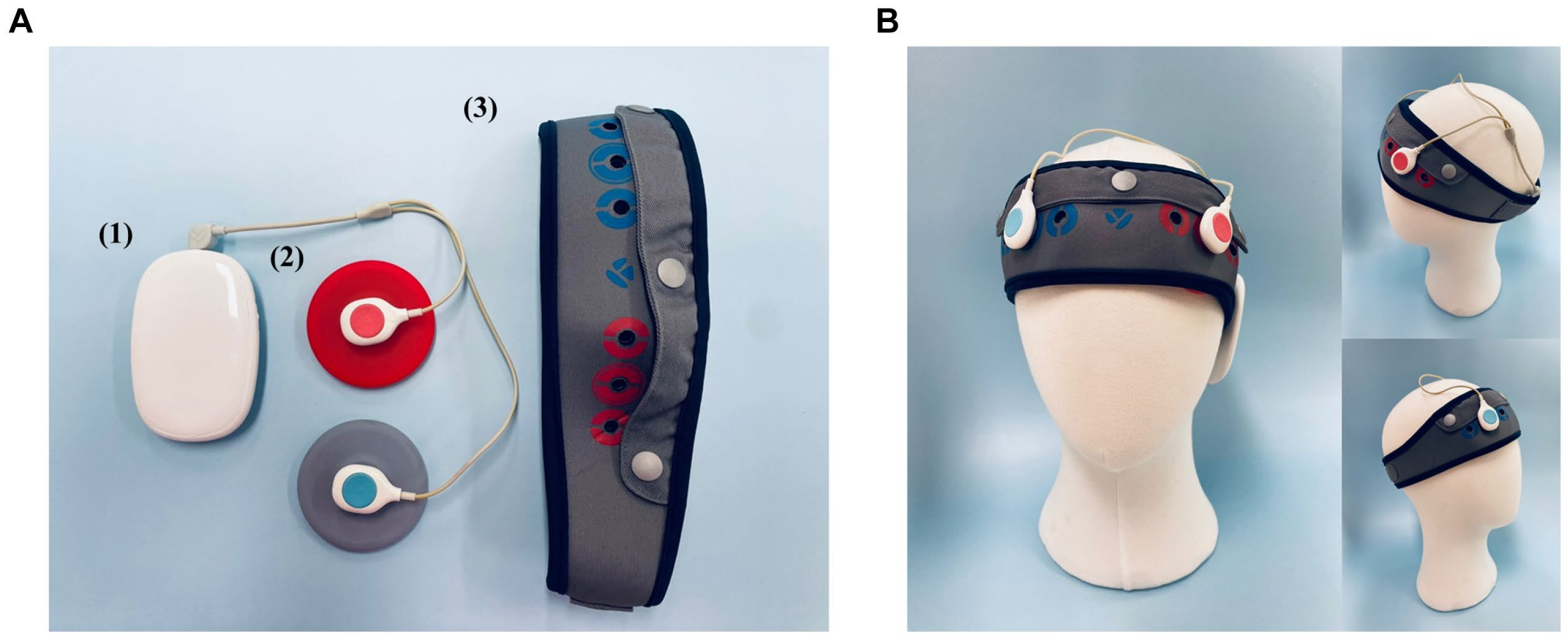
The tDCS Device (YMS-201B+, Ybrain Inc., Seongnam, Korea). **(A)** (1) Module: source of electrical stimulation. (2) Electrode: the anode is red; the cathode is blue. The diameter of the electrode is 6 cm (area 28.3 cm^2^). (3) Head band: the position of the electrode can be personalized. The fixing hole where the electrode is fixed can be located anywhere by perforating a hole. The chin strap is integrated with the head cap to secure a stable position. **(B)** The anode (positive electrode) was positioned over the left dorsolateral prefrontal cortex (dlPFC; F3), and the cathode (negative electrode) was located over the right dlPFC (F4), in accordance with the international 10–20 EEG system for electrode placement. The electrodes were initially soaked in a saline solution to ensure optimal conductivity and comfort for the participant during stimulation.

For administration of tDCS, the YMS-201B+ device (Ybrain Inc, Seongnam, Korea) was utilized (Figure 2). The anode (positive electrode) was positioned over the left dorsolateral prefrontal cortex (dlPFC; F3) and the cathode (negative electrode) was located over the right dlPFC (F4), following the international 10–20 EEG system for electrode placement. The electrodes were initially soaked in saline solution to ensure optimal conductivity and comfort for the participant during stimulation. The intensity was set to 2 mA, and each tDCS session lasted 30 min.

Patients were randomly assigned to two groups: the Active-tDCS group receiving actual tDCS stimulation and the Sham-tDCS group receiving sham (placebo) stimulation. For the sham stimulation, the same procedure was followed as for the actual stimulation, but the device was turned off after 30 s, providing a sensation of initial stimulation but no active current for the remainder of the session. This is a common practice in tDCS studies to ensure blinding (Gandiga et al., 2006).

In consideration of safety and feasibility, patients or their caregivers were trained by the research team to administer the tDCS at home independently. Prior to the start of the study, the team provided detailed instructions and demonstrated the correct procedure for electrode placement and operation. The importance of consistent placement of the electrodes was emphasized. Patients or caregivers were instructed to clean the device and the treated area and to report any discomfort or adverse events during the trial period. To maintain adherence to the intervention protocol, regular follow-ups were conducted to monitor compliance and to answer any queries related to the procedure. This approach facilitated self-administration of the tDCS intervention and contributed to the overall feasibility of this pilot study (Charvet et al., 2020).

### 2.4 Neuropsychological tests

For assessment of cognitive function, the Seoul Neuropsychological Screening Battery (SNSB) was employed (Ryu and Yang, 2023). Attention and working memory were gauged through the digit span test, which includes both forward and backward components (Leung et al., 2011). Language abilities were appraised using the Korean version of the Boston Naming Test (K-BNT) (Kim and Na, 1999). Visuospatial capacities were evaluated based on the copy score derived from the Rey Complex Figure Test (RCFT) (Meyers and Meyers, 1995). To understand verbal and visual memory, scores from the Seoul Verbal Learning Test (SVLT) and the RCFT were utilized (Kwak and Cho, 2004). The immediate recall scores were determined as the sum of scores from three attempts, and the delayed recall and recognition scores from both tests were also considered. When examining the functions related to the frontal lobe, the Controlled Oral Word Association Test (COWAT) was utilized (Kang et al., 2000), considering its two semantic word fluency scores (categories: animals, supermarket) and the cumulative scores of three phonemic word fluency tasks based on Korean phonemes: ㄱ, ㅇ, and ㅅ. Additionally, the Korean-Color Word Stroop Test (K-CWST) provided insights, especially through its color reading score (Lee et al., 2000). The neuropsychological evaluations were performed twice: once at baseline, followed by an interval of 12 weeks of tDCS application performed at home, and a second time between the 13th and 14th weeks after baseline to ascertain the immediate impacts of the tDCS intervention.

### 2.5 Blood sampling and plasma Aβ oligomer assay

To assess the presence of oligomeric beta-amyloid, a detailed procedure utilizing the Multimer Detection System (MDS) technique, which operates similarly to an Enzyme-Linked Immunosorbent Assay (ELISA), was implemented (An et al., 2010, 2017). Venous blood was drawn at 10 mL and collected into sodium heparin-containing tubes that were centrifuged at 850 × *g* for 30 min. Subsequently, the plasma supernatant was extracted, aliquoted, and stored in polypropylene tubes of 0.5 mL capacity at −80°C, pending analysis.

The 6E10 antibody, a monoclonal mouse antibody (BioLegend, San Diego, CA, USA) at a concentration of 3 μg/mL in a carbonate–bicarbonate buffer (pH 9.6), was applied to coat a 96-well black plate overnight at 4°C. This was followed by a 2-h blocking step at room temperature using 0.4% Block Ace (AbD Serotec, Raleigh, NC, USA). To enhance the assay’s specificity for Aβ oligomers, a pre-incubation step with 4.04 μL of heterophilic blocking reagent-1 (PBR-1) from Scantibodies Laboratory, Santee, CA, USA, was included. This step, combined with the subsequent three washes with phosphate-buffered saline (PBS), reduces non-specific binding to monomeric and fibrillar forms of Aβ and APP. The plate was then stored at 4°C until needed. On the day of the assay, plasma samples were gently thawed at 37°C over 15 min. To these samples, PBS with Tween 20 (PBST) was added, ensuring thorough mixing. The assay conditions, including a 144-h incubation period, were optimized to favor the binding of 6E10 to Aβ oligomers. Plasma samples and control solutions (100 μL) were then pipetted into the wells. Following a one-hour incubation at room temperature, the wells underwent three washes with Tris-buffered saline containing Tween 20 (TBST). Subsequently, FF51-HRP in TBST supplemented with 0.4% Block Ace was added, and the mixture was incubated for another hour at ambient temperature. To amplify detection sensitivity, particularly for oligomeric forms of Aβ, each well received 100 μL of an enhanced chemiluminescent substrate. Luminescent reactions were measured using a Victor 3 luminometer (PerkinElmer, Waltham, MA, USA). The data gathered from the luminescent reactions are presented in relative luminescence units (RLU), which typically correlate with the concentration of the analyte in the sample (Babapour Mofrad et al., 2021). The plasma AβO level was measured twice for each participant, at baseline before the application of tDCS and after 12 weeks of treatment, coinciding with the timing of the neuropsychological tests.

### 2.6 Statistical analysis

All statistical analyses were conducted using SPSS version 18.0. A two-tailed test was employed with a significance level set at 0.05. Continuous variables were analyzed using the Mann–Whitney *U* test, while categorical data were compared using the chi-square test. The assessment of efficacy was based on a comparison of evaluations at baseline and after 12 weeks. For these comparisons, a repeated measure general linear model (GLM) was utilized.

## 3 Results

### 3.1 Demographic characteristics of the participants

In the present study, 16 participants were enrolled, with eight individuals assigned to the Active-tDCS group and eight to the Sham-tDCS group. Comparative analysis revealed no significant differences between the two groups in terms of age, gender, years of education, and clinical dementia stages. Likewise, no significant disparities were found in scores of K-MMSE, CDR, and geriatric depression scale (GDS) between the groups (Table 1).

**Table 1:** Demographic characteristics between sham-tDCS and active-tDCS groups.

	Sham-tDCS	Active-tDCS	Value of *p*^†^
Number (female)	8 (4)	8 (3)	
Age (years)	69.9 ± 8.3	71.1 ± 6.6	0.437
Onset age (years)	68.4 ± 5.1	69.9 ± 5.3	0.521
Duration (months)	13.5 ± 3.4	14.4 ± 1.3	0.439
Education	11.1 ± 2.5	11.3 ± 1.7	0.487
K-MMSE	22.7 ± 3.5	23.5 ± 2.8	0.338
CDR	0.8 ± 1.9	0.8 ± 3.9	0.598
SOB	3.4 ± 3.7	3.2 ± 2.1	0.344
Barthel index	20.0 ± 0.0	20.0 ± 0.0	0.766
GDS	3.6 ± 3.4	4.1 ± 2.5	0.286

Values represent mean ± SD.

† Mann–Whitney *U* test was performed. K-MMSE, Korean Mini-Mental State Examination; CDR, Clinical Dementia Rating Scale; SOB, Sum of Boxes; GDS, Geriatric Depression Scale.

### 3.2 Changes in neuropsychological functioning post-tDCS interventions

The Active-tDCS group exhibited a noteworthy enhancement in the K-MMSE score from a baseline of 23.2 ± 2.3 to 25.60 ± 1.6 post-intervention, with a significant time × group interaction (*p* = 0.036). Similarly, there was a meaningful decrease in the CDR score from 0.8 ± 1.9 to 0.52 ± 0.2, again marked by a notable interaction effect (*p* = 0.028).

For the neuropsychological assessments, the Active-tDCS group showed significant progress in several domains. The digit forward span increased from 3.70 ± 0.5 to 4.40 ± 1.0 with a consequential interaction effect (*p* = 0.049), and the digit backward span increased from 2.60 ± 0.8 to 3.90 ± 1.3 (*p* = 0.038). The Go-no-go test scores in the Active-tDCS group improved from 11.80 ± 4.9 to 16.69 ± 4.4, with an interaction effect (*p* = 0.008). The K-BNT scores increased from 43.20 ± 7.8 to 49.00 ± 6.5 (*p* = 0.047). The Stroop tests in word and color, as well as test of the calculation domain demonstrated better scores in the post-intervention phase with evident interaction effects. Although there was an improvement in immediate recall in the language domain, no significant difference was noted for visual memory compared to the Sham-tDCS group.

Conclusively, the Active-tDCS group outcomes indicated prominent enhancements, particularly in areas of language ability, verbal memory, attention span, and frontal lobe-related functions. The observed improvements were consistently backed by significant time × group interaction effects across various neuropsychological evaluations (Table 2).

**Table 2:** A comparison of neuropsychological tests between sham-tDCS and active-tDCS groups at baseline (0 week) and post-tDCS intervention (12 weeks).

		At baseline (0 week)		Post-tDCS intervention (12 weeks)		Value of *p*^†^
		Sham-tDCS	Active-tDCS	Sham-tDCS	Active-tDCS	
DST	Forward	3.80 ± 0.9	3.70 ± 0.5	3.70 ± 0.9	4.40 ± 1.0	0.049
	Backward	3.10 ± 1.3	2.60 ± 0.8	3.04 ± 0.9	3.90 ± 1.3	0.038
K-BNT		44.63 ± 8.2	43.20 ± 7.8	44.38 ± 8.7	49.00 ± 6.5	0.047
Calculation		11.70 ± 0.6	11.00 ± 0.9	11.30 ± 0.9	11.70 ± 0.6	0.019
Ideomotor praxis		4.50 ± 0.5	4.40 ± 0.5	4.50 ± 0.5	4.50 ± 0.5	0.816
SVLT	Immediate recall	13.79 ± 6.1	13.20 ± 3.7	13.60 ± 5.3	16.30 ± 6.0	0.041
	Delayed recall	3.89 ± 2.3	3.20 ± 2.2	3.50 ± 2.9	4.39 ± 3.1	0.154
RCFT	Copy	32.15 ± 6.3	32.40 ± 7.7	32.55 ± 6.5	34.65 ± 10.3	0.632
	Immediate copy	10.70 ± 8.5	9.70 ± 7.1	11.00 ± 9.6	10.40 ± 8.5	0.423
	Delayed copy	9.45 ± 8.3	8.40 ± 7.2	10.35 ± 10.3	10.05 ± 8.5	0.549
Contrasting		20.00 ± 0.0	20.00 ± 0.0	20.00 ± 0.0	19.80 ± 0.6	0.331
Go-no-go		14.30 ± 5.3	11.80 ± 4.9	13.50 ± 5.4	16.69 ± 4.4	0.008
Fist-edge-arm		1.25 ± 0.4	1.67 ± 0.5	1.25 ± 0.4	1.50 ± 0.5	0.678
Alternating	Hand	2.30 ± 0.9	2.20 ± 1.4	2.21 ± 1.1	2.20 ± 1.1	0.532
	Square	1.53 ± 1.1	1.55 ± 1.3	1.47 ± 1.4	1.48 ± 1.6	0.735
Luria		1.59 ± 2.8	1.62 ± 3.7	1.60 ± 1.1	1.62 ± 4.3	0.639
COWAT	Animal	12.80 ± 3.9	10.40 ± 2.7	11.80 ± 4.3	11.90 ± 3.2	0.147
	Supermarket	12.81 ± 7,2	10.40 ± 4.0	13.40 ± 5.0	12.20 ± 3.3	0.515
	Phonemic	21.60 ± 11.5	16.00 ± 4.1	23.59 ± 12.2	21.69 ± 8.2	0.142
CWST	Word correct	107.2 ± 7.1	102.60 ± 11.7	107.60 ± 7.2	108.80 ± 8.6	0.039
	Color correct	47.95 ± 26.7	46.25 ± 51.4	41.58 ± 23.7	51.58 ± 23.7	0.048
K-MMSE		22.7 ± 3.5	23.2 ± 2.3	22.2 ± 3.1	25.60 ± 1.6	0.036
CDR		0.8 ± 1.9	0.8 ± 1.9	0.80 ± 0.2	0.52 ± 0.2	0.028
SOB		3.4 ± 3.7	3.2 ± 2.1	3.4 ± 2.36	2.8 ± 2.1	0.035
GDS		3.6 ± 3.4	4.1 ± 2.5	3.5 ± 5.1	4.0 ± 6.3	0.264

Values represent mean ± SD.

† Repeated measure general linear model (GLM) analysis was performed. DST, Digit Span Test; K-BNT, Korean Boston Naming Test; SVLT, Seoul Verbal Learning Test; RCFT, Rey-Complex Figure Test; COWAT, Controlled Oral Word Association Test; CWST, Color Word Stroop Test; K-MMSE, Korean Mini-Mental State Examination; CDR, Clinical Dementia Rating Scale; SOB, Sum of Boxes; GDS, Geriatric Depression Scale.

### 3.3 Changes in plasma Aβ oligomerization level post-tDCS intervention

**Figure 3:**
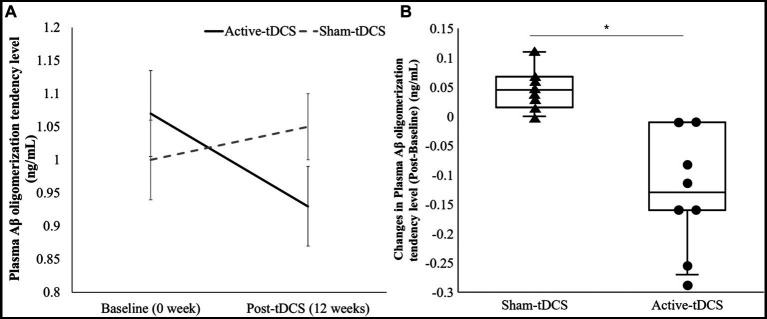
**(A)** A comparison of plasma Aβ oligomerization level between sham-tDCS and active-tDCS groups at baseline (0 week) and post-tDCS intervention (12 weeks). The lines represent mean values, and the error bars indicate the standard error. **(B)** Box plot of changes in plasma Aβ oligomerization levels from baseline to post-tDCS. Each dot indicates the individual value. *Significant difference between the groups (*p* < 0.05).

Upon evaluating the plasma Aβ oligomerization level, significant differences were shown between the Active-tDCS and Sham-tDCS groups over 12 weeks (Figure 3). Initially, the Active-tDCS group recorded a level of 1.07 ± 0.13 ng/mL. Following the intervention, this decreased to 0.93 ± 0.12 ng/mL. Conversely, the Sham-tDCS group started with a baseline of 1.00 ± 0.12 ng/mL and showed no change post-intervention, registering a consistent 1.05 ± 0.10 ng/mL. Statistical analyses revealed a pronounced time x group interaction effect (*p* < 0.001). Moreover, the post-intervention changes in the plasma Aβ oligomerization level was significantly different between groups: −0.14 ± 0.11 ng/mL in the Active-tDCS group versus 0.05 ± 0.04 ng/mL in the Sham-tDCS group (*p* < 0.001).

## 4 Discussion

Our investigation sought to discern the potential enhancements in cognitive function and alterations in blood biomarkers following tDCS application. This study targeted patients with mild AD who were not only undergoing pharmacological treatment, but also tested positive for amyloid PET.

This study showed that application of tDCS over a period of 12 weeks significantly enhances the overall cognitive functions of patients with amyloid PET-positive AD who were already on pharmacological treatment. Notably, these patients exhibited pronounced improvements in linguistic abilities, verbal memory, attentional focus, and functions related to the frontal lobe. In contrast to the Sham-tDCS group, participants in the Active-tDCS group demonstrated a noteworthy enhancement in cognitive function, as evidenced by the significant elevation in K-MMSE scores from 23.2 ± 2.3 to 25.60 ± 1.6. Additionally, the CDR scores in the same group markedly decreased from 0.8 ± 1.9 to 0.52 ± 0.2, underscoring a reduced severity of dementia. These findings are consistent with a prior study that administered prefrontal tDCS to 34 AD patients at 2 mA for 25 min for a total of 10 sessions. That study reported a significant improvement in MMSE scores in comparison to the control group, echoing the beneficial effects observed in our research (Khedr et al., 2014). Additionally, using the same device as in our study, a study that applied tDCS at home for 6 months also observed a significant improvement in MMSE scores compared to the control group (Im et al., 2019). This suggests that a 12-week or approximately 3-month application can potentially enhance overall cognitive abilities. However, while the MMSE was originally devised as a brief screening tool, its results should be interpreted with caution, especially when considered alongside other cognitive and neuroimaging findings.

Our study is the first to report significant improvements in CDR scores, emphasizing the significance and potential impact of our findings. Such tangible advancements highlight the potential efficacy of tDCS in rehabilitating the cognitive faculties that are often compromised in AD, suggesting a promising avenue for therapeutic strategies targeting specific regions of the brain.

In the detailed subtests of neuropsychological assessment, significant improvements were observed in the Active-tDCS group compared to the Sham-tDCS group. Specifically, the DST results evaluating attention abilities in both forward and backward tasks, the K-BNT results assessing language capabilities, and findings from the Go-No Go test and CWST, which assess executive functions, all showed notable enhancements in the Active-tDCS group. The left dorsolateral prefrontal cortex (DLPFC), targeted by our tDCS protocol, plays a central role in attention, language, and executive functions (Hertrich et al., 2021; Sanchez-Lopez et al., 2021). The DLPFC proximity to Broca’s area suggests that stimulating it can enhance language abilities, as reflected in the improved K-BNT results in our Active-tDCS group. Additionally, the frontal lobe, which houses the DLPFC, is crucial for executive functions such as planning and problem-solving. Our findings from the DST forward and backward tests further indicate that tDCS may bolster both attentional and executive processes. These cognitive gains might be attributed to the potential of tDCS in promoting neuroplasticity and cortical excitability.

The fundamental mechanism of tDCS involves delivering a weak current to specific areas of the brain, modulating neuronal excitability. Anodal tDCS decreases the threshold for neuronal activity, enhancing activation, while cathodal tDCS increases this threshold, reducing neuronal activation. This modulation significantly affects neurotransmitter systems, altering levels of key neurotransmitters like dopamine, serotonin, and glutamate, thereby influencing cognitive functions and mood regulation (Nitsche et al., 2015). However, the precise mechanisms of tDCS in dementia, particularly in AD, are not fully understood. Especially, the impact of tDCS on cognitive decline associated with amyloid-beta accumulation remains especially uncertain. Therefore, further research is essential to elucidate the functioning of tDCS in AD models. This should include in-depth studies on the changes in neurotransmitters related to amyloid-beta, synaptic plasticity, and brain circuitry alterations. Such investigations are vital to understand how tDCS might contribute to AD treatment strategies. Importantly, our study, although presenting preclinical results, is notable as it is the first to assess changes in plasma Aβ oligomerization levels pre- and post-tDCS application in mild AD patients confirmed positive for amyloid PET.

Compared to the Sham-tDCS group, the Active-tDCS group showed a notable reduction in post-stimulation plasma Aβ oligomerization level (Changes: Active-tDCS, −0.14 ± 0.11 ng/mL vs. Sham-tDCS, 0.05 ± 0.04 ng/mL). Monitoring amyloid-beta level pre- and post-intervention in mild dementia patients is crucial for assessing treatment efficacy and understanding pathological changes (DaSilva et al., 2010). Previous research indicated the role of tDCS in preventing glucose metabolism decreases in the middle/inferior temporal gyrus (Im et al., 2019) but did not address amyloid-beta level changes. In animal studies exploring the neurovascular unit (NVU) enhancement mechanism of tDCS in AD, Luo et al. (2022) examined its impact on mice in prodromal stages. They found significant metabolic changes in β-amyloid post-tDCS, with notable β-amyloid, amyloid precursor protein, and BACE1 reductions in the prefrontal cortex and hippocampus of the stimulated group. Concurrently, ADAM10 level increased. The study also observed improvements in cerebral neurovascular density and vessel length, reduced IgG extravasation, and increased occludin protein level, suggesting the tDCS potential to boost NVU function in early-stage AD APP/PS1 mice. The NVU, a complex of neurons, glial cells, and cerebral blood vessels, plays a central role in Aβ clearance, especially through glial cells and blood vessels (Zlokovic, 2010). Astrocytes aid in Aβ clearance by disrupting degrading enzymes and receptors, while microglia contribute through phagocytosis (Apátiga-Pérez et al., 2022). Recent studies on tDCS effects on astrocytes and blood vessels have unveiled potential connections. Our preliminary results suggest tDCS may bolster NVU activity, especially in astrocytes and blood vessels, enhancing Aβ clearance. However, these are initial findings, necessitating further validation in future research. Recent advancements spotlight plasma Aβ oligomer level measurements as a non-invasive, cost-effective method for evaluating therapeutic interventions in mild dementia, offering an alternative to invasive methods like CSF Aβ42 concentration or amyloid PET imaging.

In this study, we targeted the left dlPFC using bifrontal tDCS to improve cognitive functions in patients with mild AD. Over a period of 12 weeks, this approach significantly enhanced overall cognitive functions, as indicated by improvements in MMSE and GDS scores, and executive abilities associated with the dlPFC, such as DST. Additionally, we observed a positive impact on Aβ clearance. Complementing our findings, other studies have investigated the application of tDCS in the temporal and occipital lobes for dementia (Cotelli et al., 2014; Barbieri et al., 2016). For example, Cotelli et al. (2014) focused on the temporal lobe to enhance language comprehension, reporting positive effects on language processing. In a similar vein, Barbieri et al. (2016) applied tDCS to the occipital lobe, resulting in improved visual cognitive functions. These studies collectively underscore the versatility of tDCS in targeting different brain regions to alleviate specific symptoms of dementia. However, despite these encouraging results, the field still necessitates further research for a more profound understanding and the development of more efficacious treatment methodologies. In particular, additional studies are imperative to investigate changes at the cellular level, including Aβ clearance and neurotransmitter modifications, to comprehensively understand the impact of tDCS on dementia.

While our study offers valuable insights, it has certain limitations. First, similar to many studies in this field, our small sample size limits the statistical robustness of our conclusions. Second, we focused on the short-term effects of tDCS. To truly understand its efficacy, it is imperative to assess its impact over time: post-intervention, at 6 months, and also at extended milestones like the 1- and 2-year marks. Such prolonged evaluations are crucial to determine the long-term impacts and its potential preventive effects on dementia progression. Moreover, the reliability of our findings regarding changes in oligomer beta-amyloid would be supported by larger studies. Comparing our results with established beta-amyloid measures, such as CSF and FDG-PET, would enhance their validity. The exact mechanisms by which tDCS influences plasma beta-amyloid concentration also warrant further exploration. Finally, our focus on mild dementia patients undergoing drug treatment for beta-amyloid positivity opens doors for more extensive research, including tDCS effects across dementia stages and types and its potential combined benefits with cognitive rehabilitation.

## 5 Conclusion

This investigation sheds light on the potential therapeutic benefits of tDCS in patients with amyloid PET positivity who are already on pharmacological treatment. Our findings underscore the capacity of tDCS, applied over a span of 12 weeks, to notably improve cognitive faculties – particularly linguistic abilities, verbal memory, attentional focus, and frontal lobe functions. Furthermore, our findings on plasma Aβ oligomerization level post-tDCS administration spotlight intriguing biomarker shifts that deserve further exploration. In conclusion, while the advancements gleaned from our study signal an exciting and promising avenue in AD therapeutic interventions, much more data are needed. The potential synergies of tDCS with other treatments, its efficacy across dementia stages and types, and the possibilities it holds when paired with cognitive rehabilitation endeavors should be determined. As the global burden of dementia increases, interventions like tDCS could pave the way for more holistic and effective therapeutic strategies.

## Data availability statement

The raw data supporting the conclusions of this article will be made available by the authors, without undue reservation.

## Ethics statement

The studies involving humans were approved by Institutional Review Board of Soonchunhyang University Cheonan Hospital. The studies were conducted in accordance with the local legislation and institutional requirements. Written informed consent for participation in this study was provided by the participants’ legal guardians/next of kin.

## Author contributions

JK: Conceptualization, Formal analysis, Methodology, Visualization, Writing – original draft. YY: Conceptualization, Funding acquisition, Investigation, Methodology, Project administration, Resources, Supervision, Writing – review & editing, Validation.
